# Key to the females of Afrotropical *Anopheles* mosquitoes (Diptera: Culicidae)

**DOI:** 10.1186/s12936-020-3144-9

**Published:** 2020-02-13

**Authors:** Maureen Coetzee

**Affiliations:** 1 Wits Research Institute for Malaria, School of Pathology, Faculty of Health Sciences, University of the Witwatersrand, Johannesburg, South Africa; 2 Centre for Emerging Zoonotic & Parasitic Diseases, National Institute for Communicable Diseases, Johannesburg, South Africa

**Keywords:** *Anopheles*, Morphology, Identification, Key

## Abstract

**Background:**

In 1987, Gillies and Coetzee published a pictorial key for the morphological identification of adult female mosquitoes. Since then, several new species of anopheline mosquitoes have been described.

**Methods:**

The 1987 key to adult female mosquitoes was used as the template for the current key.

**Results:**

New species described in the literature over the past 32 years have been included. A list of all currently known Afrotropical species is provided. *Anopheles stephensi* is included for the first time as occurring on the African continent.

**Conclusions:**

An updated key for the morphological identification of Afrotropical anopheline species is presented.

## Background

Dichotomous keys for the morphological identification of groups of organisms have been used for over 300 years. These keys lead the reader through a series of couplets, each one giving two choices of characters, until a species name is reached. For the anopheline mosquitoes of the Afrotropical Region, which includes some of the most efficient transmitters of malaria parasites in the world, the first key for their identification was published in 1931 [1], and the most recent printed version in 1987 [2], this last being a pictorial key containing line graphics of characters mentioned in each couplet. In the 32 years that have passed since Gillies and Coetzee published their key [2], several new species have been discovered, described and named.

## Methods

The pictorial key to adult female anophelines in the Afrotropical Region [2] was used as the template for the current key. New illustrations were produced and new couplets inserted where appropriate to accommodate new species described since 1987.

## Results

The present key is a revision of that presented in Gillies and Coetzee [2], with the addition of newly described species and the exclusion of subspecies. Table 1 provides a lists of species, authorship and current classification, while Table 2 gives the number of species described per decade since 1900. The user is encouraged to refer to both the 1968 volume of Gillies and De Meillon [3] and the 1987 supplement of Gillies and Coetzee for full species descriptions, biology and geographic distribution. More recent references include Sinka et al. [4], and Kyalo et al. [5].

**Table 1:** List of the species of *Anopheles* (excluding subspecies) in the Afrotropical Region, excluding Madagascar and associated islands

Subgenus	Series	Section	Group/complex*	Species	Authors
*Anopheles*	Myzorhynchus		Coustani	*caliginosus*	De Meillon, 1943
			Coustani	*coustani*	Laveran, 1900
			Coustani	*crypticus*	Coetzee, 1994
			Coustani	*namibiensis*	Coetzee, 1984
			Coustani	*paludis*	Theobald, 1900
			Coustani	*symesi*	Edwards, 1928
			Coustani	*tenebrosus*	Dönitz, 1902
			Coustani	*ziemanni*	Grünberg, 1902
				*obscurus*	(Grünberg, 1905)
	Anopheles			*concolor*	Edwards, 1938
*Cellia*	Neomyzomyia	Smithii		*caroni*	Adam, 1961
				*faini*	Leleup, 1952
				*hamoni*	Adam, 1962
				*jebudensis*	Froud, 1944
				*lovettae*	Evans, 1934
				*rageaui*	Mattingly & Adam, 1954
				*smithii*	Theobald, 1905
				*vanhoofi*	Wanson & Lebied, 1945
				*wilsoni*	Evans, 1934
		Ardensis		*ardensis*	(Theobald, 1905)
				*buxtoni*	Service, 1958
				*cinctus*	(Newstead & Carter, 1910)
				*dualaensis*	Brunhes, Le Goff & Geoffroy, 1999
				*deemingi*	Service, 1970
				*dureni*	Edwards, 1938
				*eouzani*	Brunhes, Le Goff & Boussès, 2003
				*kingi*	Christophers, 1923
				*machardyi*	Edwards, 1930
				*maliensis*	Bailly-Choumara & Adam, 1959
				*millecampsi*	Lips, 1960
				*multicinctus*	Edwards, 1930
				*natalensis*	(Hill & Haydon, 1907)
			Nili	*carnevalei*	Brunhes, Le Goff & Geoffroy, 1999
			Nili	*nili*	(Theobald, 1904)
			Nili	*ovengensis*	Awono-Ambene, Kengne, Simard, Antonio-Nkondjio & Fontenille, 2004
			Nili	*somalicus*	Rivola & Holstein, 1957
				*vernus*	Gillies & De Meillon, 1968
				*vinckei*	De Meillon, 1942
		Rhodesiensis		*cameroni*	De Meillon & Evans, 1935
				*lounibosi*	Gillies & Coetzee, 1987
				*rhodesiensis*	Theobald, 1901
				*rodhaini*	Leleup & Lips, 1950
				*ruarinus*	Edwards, 1940
	Myzomyia			*azaniae*	Bailly-Choumara, 1960
				*barberellus*	Evans, 1932
				*bervoetsi*	D’Haenens, 1961
				*brunnipes*	(Theobald, 1910)
				*domicolus*	Edwards, 1916
				*dthali*	Patton, 1905
				*erythraeus*	Corradetti, 1939
				*ethiopicus*	Gillies & Coetzee, 1987
				*flavicosta*	Edwards, 1911
				*fontinalis*	Gillies & De Meillon, 1968
				*gabonensis*	Rahola, Makanga & Paupy, 2014
				*moucheti*	Evans, 1925
				*schwetzi*	Evans, 1934
				*tchekedii*	De Meillon & Leeson, 1940
				*walravensi*	Edwards, 1930
		Funestus	Funestus	*aruni*	Sobti, 1968
			Funestus	*funestus*	Giles, 1900
			Funestus	*funestus*-*like*	(See Spillings et al. [26])
			Funestus	*parensis*	Gillies, 1962
			Funestus	*vaneedeni*	Gillies & Coetzee, 1987
			Rivulorum	*brucei*	Service, 1960
			Rivulorum	*fuscivenosus*	Leeson, 1930
			Rivulorum	*rivulorum*	Leeson, 1935
			Rivulorum	*rivulorum*-*like*	(see Cohuet, et al. [27])
				*confusus*	Evans & Leeson, 1935
				*culicifacies*	Giles, 1901
				*leesoni*	Evans, 1931
				*longipalpis*	(Theobald, 1903)
		Marshallii-Hancocki		*austenii*	(Theobald, 1905)
				*berghei*	Vincke & Leleup, 1949
				*brohieri*	Edwards, 1929
				*gibbinsi*	Evans, 1935
				*hancocki*	Edwards, 1929
				*hargreavesi*	Evans, 1927
				*harperi*	Evans, 1936
			Marshallii	*hughi*	Lambert & Coetzee, 1982
			Marshallii	*kosiensis*	Coetzee, Segerman & Hunt, 1987
			Marshallii	*letabensis*	Lambert & Coetzee, 1982
			Marshallii	*marshallii*	(Theobald, 1903)
				*mortiauxi*	Edwards, 1938
				*mousinhoi*	De Meillon & Pereira, 1940
				*njombiensis*	Peters, 1955
				*seydeli*	Edwards, 1929
		Wellcomei		*distinctus*	(Newstead & Carter, 1911)
				*erepens*	Gillies, 1958
				*theileri*	Edwards, 1912
				*wellcomei*	Theobald, 1904
		Demeilloni		*carteri*	Evans & De Meillon, 1933
				*demeilloni*	Evans, 1933
				*freetownensis*	Evans, 1925
				*garnhami*	Edwards, 1930
				*keniensis*	Evans, 1931
				*lloreti*	Gil Collado, 1935
				*sergentii*	(Theobald, 1907)
	Pyretophorus			*christyi*	(Newstead & Carter, 1911)
				*daudi*	Coluzzi, 1958
			Gambiae	*amharicus*	Hunt, Wilkerson & Coetzee, 2013
			Gambiae	*arabiensis*	Patton, 1905
			Gambiae	*bwambae*	White, 1985
			Gambiae	*coluzzii*	Coetzee & Wilkerson, 2013
			Gambiae	*fontenillei*	Barrón, Paupy, Rahola, Akone-Ella, Ngangue, Wilson-Bahun, Pombi, Kengne, Costantini, Simard, González & Ayala, 2019
			Gambiae	*gambiae*	Giles, 1902
			Gambiae	*quadriannulatus*	(Theobald, 1911)
			Gambiae	*melas*	Theobald, 1903
			Gambiae	*merus*	Donitz, 1902
	Paramyzomyia			*azevedoi*	Ribeiro, 1969
				*cinereus*	Theobald, 1901
				*listeri*	De Meillon, 1931
				*multicolor*	Cambouliu, 1902
				*seretsei*	Abdulla-Khan, Coetzee & Hunt, 1998
				*turkhudi*	Liston, 1901
	Neocellia			*dancalicus*	Corradetti, 1939
				*hervyi*	Brunhes, Le Goff & Geoffroy, 1999
				*maculipalpis*	Giles, 1902
				*pretoriensis*	(Theobald, 1903)
				*rufipes*	(Gough, 1910)
				*salbaii*	Maffi & Coluzzi, 1958
				*stephensi*	Liston, 1901
	Cellia			*argenteolobatus*	(Gough, 1910)
				*brumpti*	Hamon & Rickenbach, 1955
				*cristipalpis*	Service, 1977
				*cydippis*	De Meillon, 1931
				*murphyi*	Gillies & De Meillon, 1968
				*pharoensis*	Theobald, 1901
				*squamosus*	Theobald, 1901
				*swahilicus*	Gillies, 1964
Christya**				*implexus*	(Theobald, 1903)
				*okuensis*	Brunhes, Le Goff & Geoffroy, 1997

*Anopheles gambiae, An. nili and *An. marshallii* are referred to as complexes, while *An. coustani, An. funestus* and *An. rivulorum* are groups

**Previously a Series in the subgenus *Anopheles*, *Christya* was elevated to subgeneric status by Harbach and Kitching in 2016 [28]

**Table 2:** Number of species of Afrotropical *Anopheles* described per decade since 1900

Decade	No. species	Author/co-author (number of species authored/co-authored)
1900–1910	35	Theobald (18), Giles (3), Donitz (2), Gough (2), Grunberg (2), Liston (2), Patton (2), Cambouliu (1), Carter (1), Haydon (1), Hill (1), Laveran (1), Newstead (1)
1911–1920	6	Edwards (3), Carter (2), Newstead (2), Theobald (1)
1921–1930	13	Edwards (9), Evans (2), Christophers (1), Leeson (1)
1931–1940	24	Evans (12), De Meillon (6), Edwards (4), Leeson (3), Corradetti (2), Gil Collado (1), Pereira (1)
1941–1950	6	De Meillon (2), Leleup (2), Froud (1), Lebied (1), Lips (1), Vincke (1), Wanson (1)
1951–1960	13	Adam (2), Bailly-Choumara (2), Coluzzi (2), Service (2), Gillies (1), Hamon (1), Holstein (1), Leleup (1), Maffi (1), Mattingly (1), Peters (1), Rickenbach (1), Rivola (1)
1961–1970	11	Gillies (5), De Meillon (3), Adam (2), D’Haenens (1), Ribeiro (1), Service (1), Sobti (1)
1971–1980	1	Service (1)
1981–1990	8	Coetzee (7), Gillies (3), Lambert (2), Hunt (1), Segerman (1), White (1)
1991–2000	6	Brunhes (4), Le Goff (4), Geoffroy (4), Coetzee (2), Abdulla-Khan (1), Hunt (1)
2001–2010	4	Coetzee (2), Fontenille (2), Kengne (2), Simard (2), Antonio-Nkondjio (1), Awono-Ambene (1), Bousses (1), Brooke (1), Brunhes (1), Chiphwanya (1), Cohuet (1), Hunt (1), Koekemoer (1), Le Goff (1), Spillings (1), Toto (1)
2011–2020	4	Coetzee (2), Paupy (2), Rahola (2), Wilkerson (2), Akone-Ella (1), Ayala (1), Barrón (1), Costantini (1), González (1), Hunt (1), Kengne (1), Makanga (1), Ngangue (1), Pombi (1), Simard (1), Wilson-Bahun (1)

The following species are not included in the keys: *Anopheles ethiopicus* lacks hindlegs [2], *Anopheles erythraeus* and *Anopheles dualaensis* adults are unknown [3, 6], and *Anopheles eouzani* lacks hindtarsomeres 4 and 5 [7]. The following new species are included: *Anopheles okuensis* [8] (Section I), *Anopheles hervyi* [6], *Anopheles millecampsi* [8] and *Anopheles multicinctus* [9] (Section IV), *Anopheles rageaui* [6] and *Anopheles seretsei* [10] (Section VII), *Anopheles kosiensis* [11] (Section IX), *Anopheles gabonensis* [12] (Section X), and *Anopheles carnevalei* [6] and *Anopheles ovengensis* [13] (Section XI).

## Discussion

A major addition to the key is the inclusion of *Anopheles stephensi* (Section IV), the Asian malaria vector with distribution from the Middle East to China. This species was first detected on the African continent in Djibouti in September 2012 and subsequently in February 2013 [14] through to December 2017 [15]. It has also recently been found in Ethiopia in 2016 [16]. The species is similar to those belonging to the *Anopheles gambiae* complex—mosquitoes with speckled legs—but differs by having the wing with two pale spots in the 2nd main dark area of the costa and vein 1, thus being similar to *Anopheles maculipalpis* and *Anopheles pretoriensis*, from which it differs by not having hindtarsomeres 4 and 5 all pale.

There are several groups of species where morphological identification is not possible using only the adult females, either because the adults look identical or because of overlap in morphological variation. Some of these species can be identified on immature characters, thus requiring eggs or larvae [3], while others require genetical methods, such as chromosomal inversions [17] or molecular assays [18, 19]. Such groups include:

- the well-known *Anopheles gambiae* complex (*An. gambiae, Anopheles coluzzii* [20]*, Anopheles arabiensis, Anopheles quadriannulatus, Anopheles amharicus* [20]*, Anopheles fontenillei* [21]*, Anopheles bwambae, Anopheles melas, Anopheles merus*);
- the *Anopheles funestus* group (*Anopheles funestus, An. funestus*-like, *Anopheles parensis, Anopheles vaneedeni, Anopheles aruni, Anopheles confusus, Anopheles leesoni, Anopheles rivulorum, An. rivulorum*-like, *Anopheles brucei, Anopheles fuscivenosus*);
- the *Anopheles nili* complex (*An. nili, Anopheles somalicus*);
- the *Anopheles marshallii* complex (*An. marshallii, Anopheles letabensis, Anopheles hughi, Anopheles kosiensis*) and its allies *Anopheles hargreavesi, Anopheles gibbinsi* and *Anopheles mousinhoi*;
- *Anopheles squamosus/cydippis,* the former of which is known to consist of at least five chromosomal forms (Green and Hunt, unpublished data);
- *Anopheles coustani/crypticus/namibiensis* in southern Africa.

The definition of “complex”, as applied to the genus *Anopheles*, is a group of species that are virtually morphologically identical but are otherwise considered valid species. The use of the term “group” denotes species that are morphologically very similar at the adult stage but many can be distinguished at the immature stages.

Except for those of medical importance, the above list is just a small sample of species groups about which we know very little biologically in terms of feeding/resting preferences or their role in malaria parasite transmission. Basic taxonomic research, aligned with molecular analyses, is still very much needed in the Culicidae.

## Conclusions

An updated key for the morphological identification of Afrotropical anopheline species is presented. This key should be used in conjunction with earlier works giving full species descriptions, biology, medical importance and distribution.

## The key layout

Characters are presented in ‘couplets’ where two options are presented, giving two different outcomes, eventually ending at a species name. The illustration(s) for the first option of each couplet is on the left (or rarely, in the centre) and for the second option on the right. General terminology follows that of Harbach and Knight [22, 23].

**Fig. 1:**
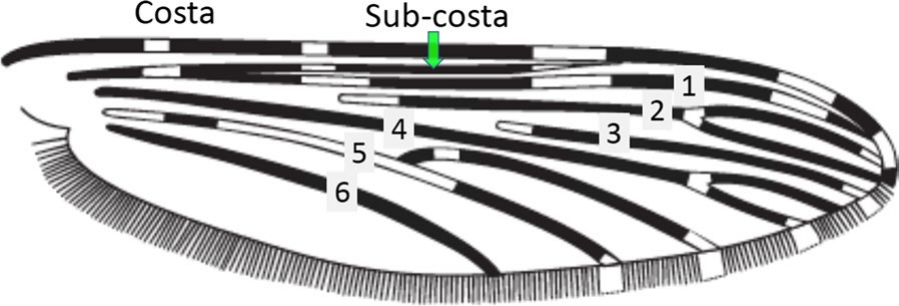
Mosquito wing showing the simplified venation numbering system of Evans [25]

Terminology of the wing venation has changed over the past 80 years in attempts to align Culicidae with the rest of the Diptera Family (Table 3). The terminology proposed in the recent Manual of Afrotropical Diptera [24] has been challenged by culicid taxonomists (manuscript reviewers), specifically around the terms used for the posterior veins (veins 5 and 6 in Fig. 1). Since consensus on the terminology has not yet been reached, and given that the malaria vector control field workers in Africa have been using the *Anopheles* identification keys published 32 years ago [2] that use the numbering shown in Fig. 1, this simplified system is used here. It avoids unnecessary repetition of the various terms in each couplet and reference can be made to Table 3 where recent terminology is required.

**Table 3:** The terminology used for wing venation since 1938 [25] is given

	Evans [25]	Gillies & De Meillon [3]; Gillies & Coetzee [2]	Snodgrass [29]	Harbach & Knight [22]	McAlpine [30]	Cumming & Wood [24]
Costa	Costa	Costa	Costa	Costa	Costa	Costa
Sub-costa	Sub-costa	Sub-costa	Sub-costa	Sub-costa	Sub-costa	Sub-costa
1	1st vein	1st vein	R_1_—1st vein	R_1_—radius-one	R_1_—anterior radius	R_1_—anterior radius
2	2.1—2nd vein upper branch 2.2—2nd vein lower branch	2nd vein upper branch 2nd vein lower branch	R_2_—2nd vein upper branch R_3_—2nd vein lower branch	R_2_—radius-two R_3_—radius-three R_2+3_—stem of vein	R_2_—upper branch of 2nd radius R_3_—lower branch of 2nd radius R_2+3_—2nd branch of radius	R_2_—upper branch of 2nd radius R_3_—lower branch of 2nd radius R_2+3_—2nd branch of radius
3	3rd vein	3rd vein	R_4+5_—3rd vein	R_4+5_—radius-four-plus-five	R_4+5_—3rd branch of radius	R_4+5_—3rd branch of radius
4	4.1—4th vein upper branch 4.2—4th vein lower branch	4th vein upper branch 4th vein lower branch	M_1+2_—4th vein upper branch M_3_—4th vein lower branch M—stem of vein	M_1_—media-one M_2_—media-two M_1+2_—stem of vein	M_1_—1st branch of media M_2_—2nd branch of media	M_1_—1st branch of media M_2_—2nd branch of media
5	5.1—5th vein upper branch 5.2—5th vein lower branch	5th vein upper branch 5th vein lower branch	Cu_1_—5th vein upper branch Cu_2_—5th vein lower branch Cu—stem of vein	M_3+4_—media-three-plus-four CuA—cubitus anterior M—stem of vein	CuA_1_—1st branch of anterior cubital CuA_2_—2nd branch of anterior cubital	M_3+4_—3rd + 4th branch of media CuA—anterior cubital
6	6th vein	6th vein	A—anal vein	1A—anal vein	A_1_—anal vein	CuP—posterior cubital

## Key to adult females

l. Abdominal segments with laterally projecting tufts of scales on segments II–VII …Section I
– Abdominal segments not so…2

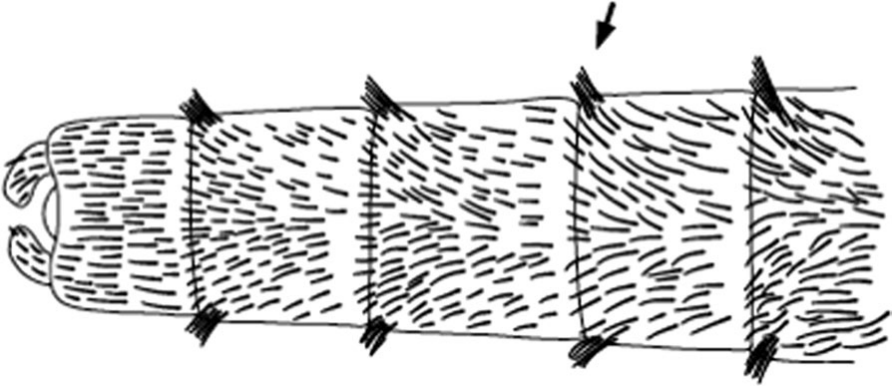

2. Hindtarsus with at least last 2 hindtarsomeres entirely pale …Section II
– Hindtarsus not so …3
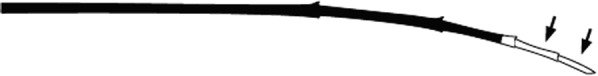
3. Hindtarsomere 5 mainly or entirely dark, hindtarsomere 4 white …Section III
– Hindtarsus not so …4
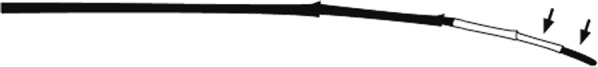
4. Legs speckled, sometimes sparsely …Section IV
– Legs not speckled …5
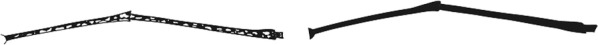
5. Wing entirely dark or with pale spots confined to costa and vein 1 …Section V
– Wing not so …6
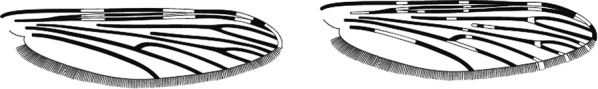
6. Wing without a pale spot on basal 0.5 of costa …Section VI
– Wing with at least 1 pale spot on basal 0.5 of costa …7
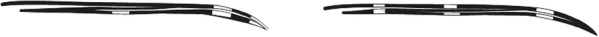
7. Maxillary palpus with apex dark …Section VII
– Maxillary palpus with apex pale …8
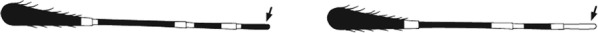
8. Maxillary palpus with 4 pale bands …Section VIII
– Maxillary palpus with less than 4 pale bands …9
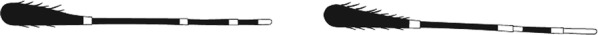
9. Wing with pale interruption in 3rd main dark area (preapical dark spot) of vein 1, sometimes fused with preceding pale area …Section IX
– 3rd main dark area without pale interruption …10
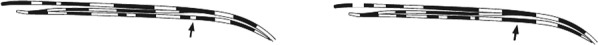
10. Wing with 2 pale spots on upper branch of vein 5 …Section X
– Wing with I pale spot on upper branch of vein 5 …Section XI
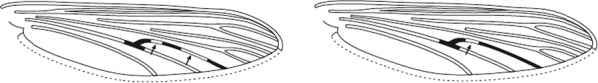

### Section I. Mosquitoes with laterally projecting tufts of abdominal scales

1. Wing almost entirely dark, costa without pale spots …*brumpti*
2. Wing with abundant pale areas, costa with at least 4 pale spots …2
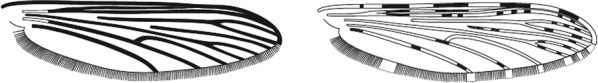
3. Hindtarsomeres 1 to 5 entirely dark … *argenteolobatus*
  (southern Africa)
  *murphyi*
  (West Africa)
4. Hindtarsomeres 1 to 4, at least, with apical pale bands …3
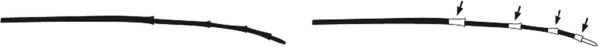
5. Hindtarsomeres 1 and 2 with definite pale and dark rings in addition to apical pale bands …*cinctus*
6. Hindtarsomeres 1 and 2 with pale bands at apices only …4
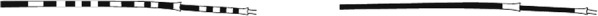
7. Hindtarsomeres 3 and 4 all white or narrowly dark basally, 5 all dark or at least basal 0.5 dark..5
8. Hindtarsomeres not so …7
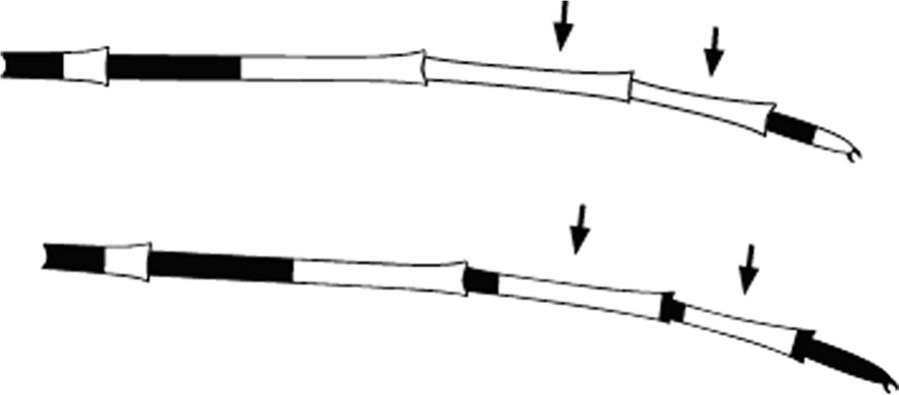
9. Moderate-sized species; abdominal scale-tufts short and dark; 0.5 or more of hindtarsomere 1 pale …*cristipalpis*
10. Very large species, abdominal segments II–VII with long lateral tufts of yellowish and dark scales; hindtarsomere 1 largely dark …6
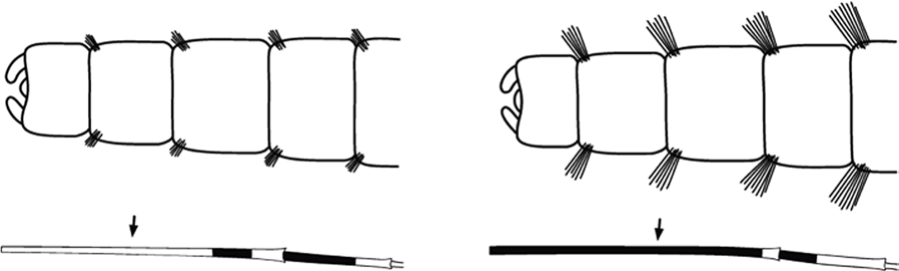
11. Pale fringe spot present opposite lower branch of vein 5 …*okuensis*
12. No pale fringe spot opposite lower branch of vein 5 …..*implexus*
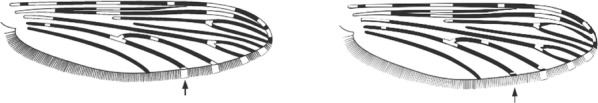
13. Hindtarsomere 5 and about apical 0.5 of 4 pale …*pharoensis*
14. Hindtarsomere 5 all dark and 4 with much less than apical 0.5 pale …8
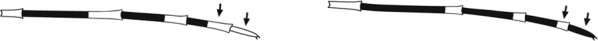
15. Very small species (wing length 2.5–2.8 mm); wing with upper branch of vein 2 largely pale …*swahilicus*
16. Small to moderate-sized species (wing length 2.7–4.5 mm); wing with upper branch of vein 2 either entirely dark apart from apex or with a few scattered pale scales only …*squamosus *
  *cydippis* 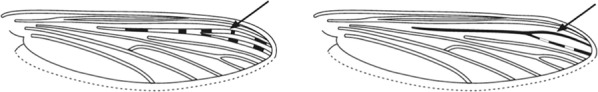

### Section II. Mosquitoes with hindtarsomeres 4 and 5 entirely white; abdomen without laterally projecting tufts of scales

1. Legs speckled …2
2. Legs not speckled …8
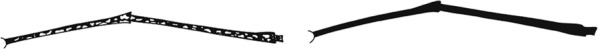
3. Hindtarsomeres 3 to 5 entirely pale …3
4. Hindtarsomere 3 dark at base …5
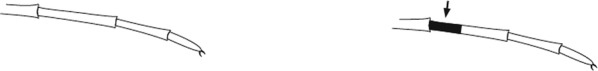
5. Maxillary palpus with 3 pale bands, usually with some speckling; vein 1 of wing with 2 pale spots in 2nd main dark area (median dark spot) … *maculipalpis*
6. Maxillary palpus with 4 pale bands, unspeckled; vein 1 of wing with at most 1 pale spot in 2nd main dark area …4
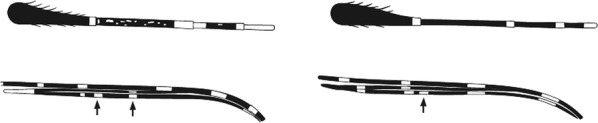
7. Midtarsomeres 2 to 4 entirely dark; vein 1 of wing dark at base, basal 0.5 of stem of vein 4 with small pale areas …*maliensis*
8. Midtarsomeres 2 to 4 with pale apices; vein 1 of wing pale at base, basal 0.5 of stem of vein 4 entirely pale …*deemingi*
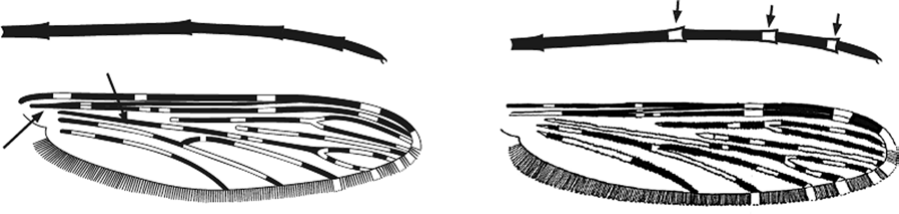
9. Hindtarsomere 1 broadly pale at apex; vein 1 of wing with 2 pale spots in 2nd main dark area …..*pretoriensis*
10. Hindtarsomere 1 narrowly pale or dark at apex; vein 1 of wing with 1 pale spot in 2nd main dark area ….6
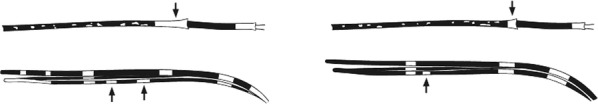
11. Foretarsomere 1 with 5–9 pale rings; stem of vein 4 of wing largely pale …*machardyi*
12. Foretarsomere 1 with 2–4 pale rings; stem of vein 4 of wing largely dark …7
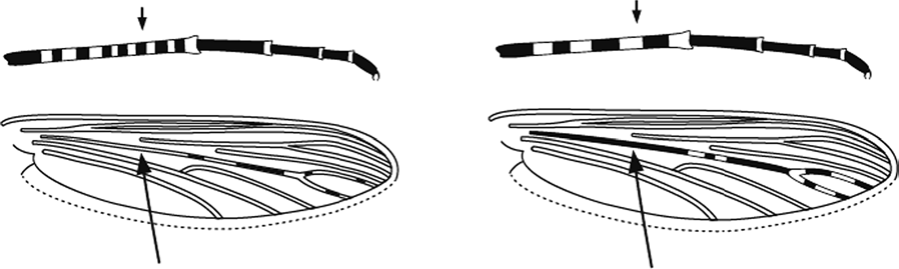
13. Fore- and midtarsomeres 2 and 3 pale at apex; wing with fringe spot opposite vein 6 …*natalensis*
14. Fore- and midtarsomeres 2 and 3 dark apically; no fringe spot opposite vein 6 …*buxtoni*
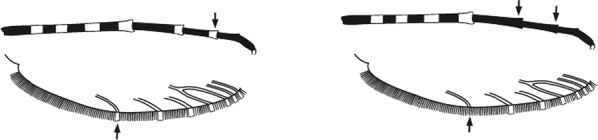
15. Maxillary palpus very shaggy and unbanded or with 1–4 irregular narrow pale bands …9
16. Maxillary palpus smooth with 3 pale bands, the 2 distal ones broad or rarely fused …14
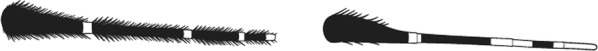
17. Maxillary palpus without pale bands; no pale spot at apex of hindtibia or base of hindtarsomere 1 …*caliginosus*
18. Maxillary palpus with 1–4 pale bands; apex of hindtibia broadly or narrowly pale …10
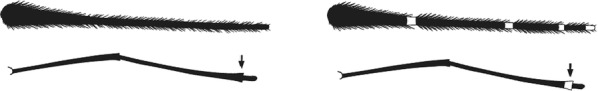
19. Hindtarsomere 3 entirely pale …11
20. Hindtarsomere 3 dark at base …12
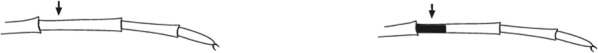
21. Base of hindtarsomere 1 dark; pale fringe spot present opposite lower branch of wing vein 5 …*paludis*
22. Base of hindtarsomere 1 broadly pale; no pale fringe spot opposite lower branch of wing vein 5 …*coustani*
  (in part)
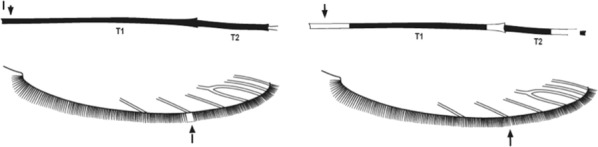
23. Hind tarsomere 1 entirely dark basally or at most with a very narrow band of pale scales not as broad as the width of the tarsomere …*tenebrosus*
  (in part)
24. Hind tarsomere 1 broadly pale at base, pale area at least as long as broad …13
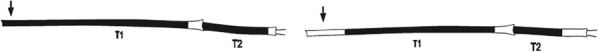
25. Apex of hindtibia with a pale streak 3–5 times as long as broad; apical pale band on hindtarsomere 2 0.13–0.4 length of tarsomere …*coustani*
  (in part)
  *crypticus*
  (S. Africa only)
26. Pale streak on hindtibia 1–3 times as long as broad; apical pale band on hindtarsomere 2 narrow, 0.07–0.13 length of segment ….*ziemanni*
  *namibiensis* 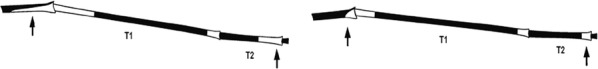

27. 3rd main dark area on wing vein 1 without a pale interruption; foretarsomeres 1 to 3 usually without distinct apical pale bands..*rufipes*
  (in part)
28. 3rd main dark area on wing vein 1 with a pale interruption, or with a short extension of the subcostal pale spot into the dark area on vein 1; foretarsomeres 1 to 3 with apical pale bands …15
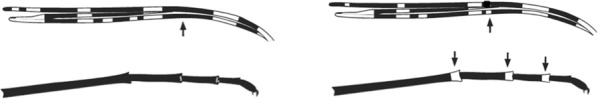
29. Hindtarsomere 3 entirely pale.…*hancocki*
  *brohieri*
  (in part)
30. Hindtarsomere 3 not so.…*brohieri*
  (in part) W. Africa
  *theileri*
  mainly E. & S. Africa
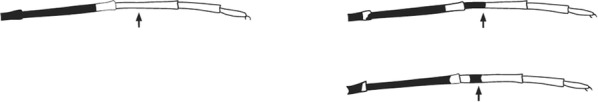

### Section III. Mosquitoes with hind tarsomere 5 mainly or entirely dark, tarsomere 4 white; abdominal segments without laterally projecting tufts of scales

1. Femora and tibiae speckled …*kingi*
2. Femora and tibiae with at most apical bands only …2
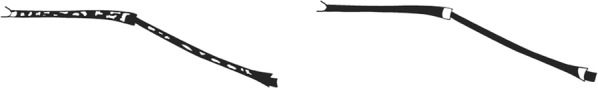
3. Maxillary palpus shaggy; costa and vein 1 of wing without usual main dark areas …*symesi*
4. Maxillary palpus smooth; 2nd main dark area of wing vein 1 well defined and with 2 pale interruptions …*rufipes*
  (in part)
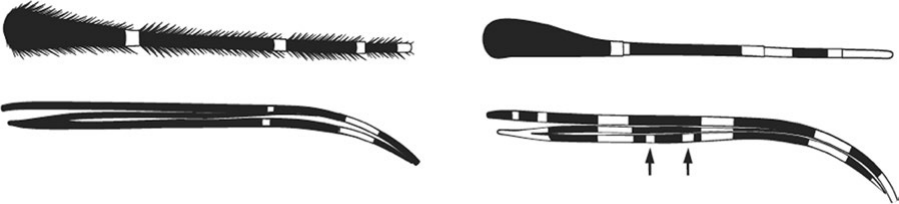

### Section IV. Mosquitoes with speckled legs, hindtarsomeres 4 and 5 not entirely pale; abdominal segments without laterally projecting tufts of scales

1. Maxillary palpus with 3 pale bands …2
2. Maxillary palpus with 4 pale bands …6
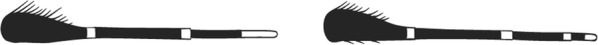
3. Maxillary palpus with apical 2 pale bands very broad, speckling on palpus segment 3; 2nd main dark area on wing vein 1 with 2 pale interruptions …*stephensi*
4. Maxillary palpus with apical dark spot about equal to or longer than apical pale band; 2nd main dark area on wing vein 1 with 1 pale interruption.…3
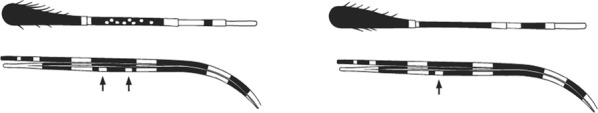
5. 3rd main dark area of wing vein 1 with a pale interruption, sometimes fused with preceding pale spot; scaling on abdomen very scanty, confined to tergum VIII or rarely VII …*gambiae* complex
  (in part)
6. 3rd main dark area of wing vein 1 without a pale interruption; abdominal terga fairly heavily clothed with cream or yellowish scales, especially on terga VI and VII …4
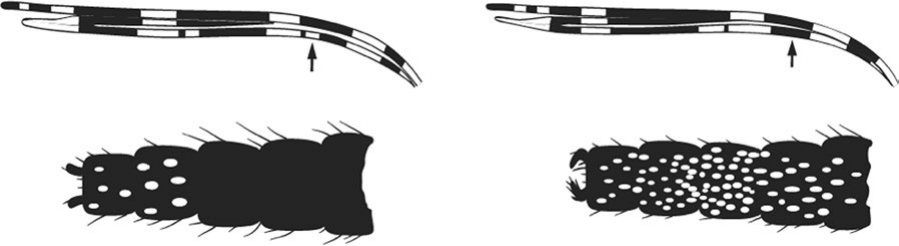
7. Maxillary palpus speckled …*hervyi*
8. Maxillary palpus not speckled ….5
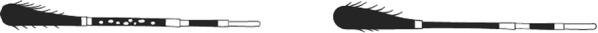
9. Foretarsomere 1 with some speckling; base of costa with 2 pale spots; stem of wing vein 2 entirely pale …*salbaii*
10. Foretarsomere 1 not speckled; base of costa with 1 pale spot; stem of wing vein 2 extensively dark …*dancalicus*
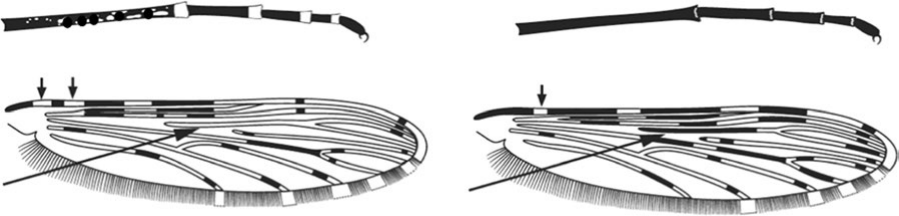
11. All tarsi completely dark; wing without pale fringe spots posterior to vein 3 …*vernus*
  (in part)
12. Tarsomeres 1 to 4 with conspicuous pale bands on at least the apices; wing with pale fringe spots up to lower branch of vein 5 or 6 …7
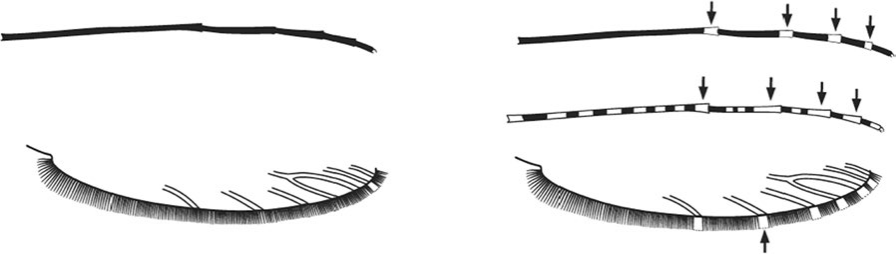
13. 3rd main dark area of vein 1 with a pale interruption, sometimes fused with preceding pale area.…*gambiae* complex
  (in part)
14. 3rd main dark area without pale interruption …8
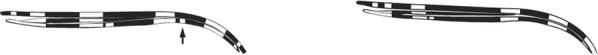
15. Hindtarsomere 2 with about apical 0.4 to 0.5 white and the rest dark …*multicinctus*
16. Hindtarsomere 2 either with less than apical 0.4 white or else prominently marked with dark and pale bands …9
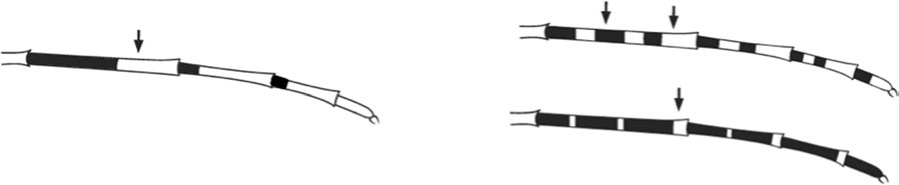
17. Hindtarsomeres 2 to 4 with apical pale rings and otherwise dark except for 1 to 2 pale spots; no pale fringe spot opposite wing vein 6 …*ardensis*
18. Hindtarsomeres 2 to 4 with conspicuous dark and pale rings in addition to apical pale bands; pale fringe spot present opposite vein 6 …10
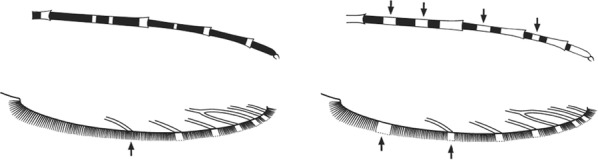
19. Foretarsomeres mainly pale with narrow dark markings ….*vinckei*
20. Foretarsomeres mainly dark with narrow pale rings …11
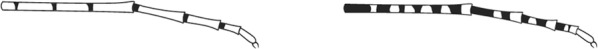
21. Scales on abdominal tergum VIII dense and distributed over whole tergum, sometimes with a few scales on lateral borders of tergum VII …*dureni*
22. Scales on tergum VIII scanty and confined to posterior margin …*millecampsi*
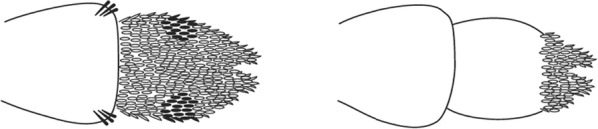

### Section V. Mosquitoes with wings entirely dark or with pale spots confined to costa and vein 1; legs not speckled, hindtarsomeres 4 and 5 not entirely pale; abdominal segments without laterally projecting tufts of scales

1. Wings entirely dark. or unicolorous …2
2. Wings with at least some areas of paler scales on costa or vein 1, these being sometimes inconspicuous …5
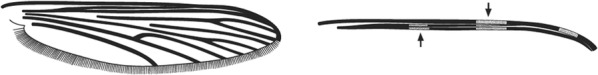
3. Maxillary palpus with 2 well-marked pale bands; hindfemur and hindtibia narrowly pale at apex …*concolor*
4. Maxillary palpus and legs entirely dark …3
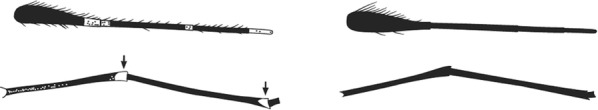
5. Large species, wing length 4 mm or more …*ruarinus*
6. Small species, wing length 3.5 mm or less …4
7. Very pale brown species with glossy scutum; semi-arid regions only ……*rhodesiensis*
  (in part)
8. General coloration dark brown, scutum not so; cave-dwelling …*caroni*
  (in part)
9. Maxillary palpus with 2 to 3 pale bands, pale at apex (sometimes indistinct) …6
10. Maxillary palpus with or without pale bands, dark at apex ….9
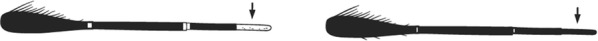
11. Erect head scales narrow, rod-like, all scales yellowish throughout; semi-arid regions only …*dthali*
12. Erect head scales broader, scales white on vertex, dark laterally; all regions ….7
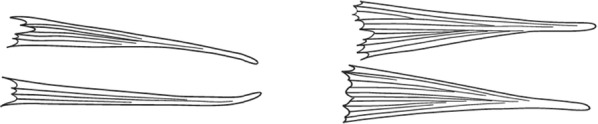
13. Pale and dark areas on wing poorly contrasted; semi-arid regions only ….*rhodesiensis*
  (in part)
14. Pale and dark areas on wing well contrasted …8
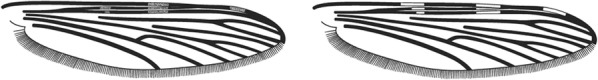
15. Pale areas on wing very narrow, subcostal pale spot present on costa only; cave-dwelling …*rodhaini*
16. Pale areas on wing broader, subcostal pale spot on costa and vein 1 ……..*rhodesiensis*
  (in part)
  *lounibosi* 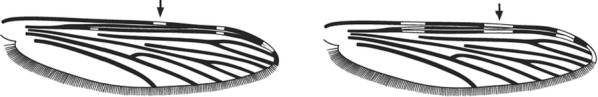

17. Maxillary palpus with 3 pale bands, dark at apex …*smithii*
  (in part)
18. Maxillary palpus unbanded or banding indistinct …10
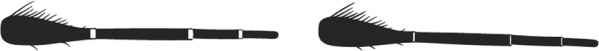
19. Cave-dwelling species; colour and contrast of dark and pale areas on wing variable …*caroni*
  (in part)
  *hamoni*
  *vanhoofi*
20. Semi-arid regions; pale brown species with poorly contrasting light and dark areas on wing ….11
21. Erect head scales narrow, rod-like, all scales yellowish throughout ….*azaniae*
  (in part)
22. Erect head scales broader, scales white on vertex, dark laterally...*rhodesiensis*
  (in part)
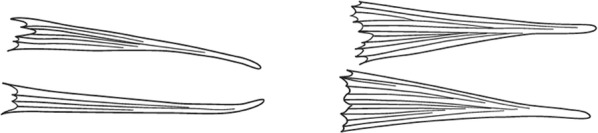

### Section VI. Mosquitoes without a pale spot on basal 0.5 of costa; pale spots not confined to costa and vein 1; legs not speckled, hindtarsomeres 4 and 5 not entirely pale; abdomen without projecting tufts of scales

1. Maxillary palpus shaggy to near tip …2
2. Maxillary palpus smooth except at extreme base ….3
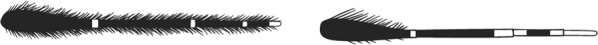
3. Maxillary palpus entirely dark; hindtarsomeres 3 and 4 dark or narrowly pale at apices …*obscurus*
  (in part)
4. Maxillary palpus with pale scales forming more or less definite pale bands; hindtarsomeres 3 and 4 narrowly or broadly pale at apices …*tenebrosus*
  (in part)
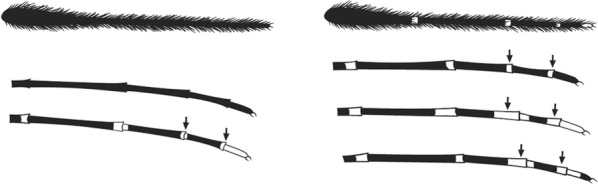
5. Maxillary palpus with apex dark, sometimes only narrowly so …..4
6. Maxillary palpus with apex pale …5
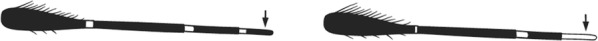
7. Stem of wing vein 4 largely pale, upper branch of vein 5 with 2 pale spots or largely pale, fringe spots present opposite vein 4 and upper branch of vein 5 …*tchekedii*
8. Stem of wing vein 4 largely dark, upper branch of vein 5 with one narrow pale area, pale fringe spots absent ...*smithii*
  (in part)
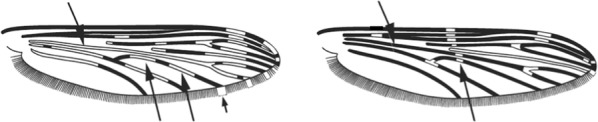
9. Costa entirely dark except for a few indistinct pale scales subapically; maxillary palpus with a broad apical pale band and otherwise dark except for a narrow basal pale band …*daudi*
10. Outer 0.5 of costa with 1–3 well-marked pale areas; maxillary palpus not so …6
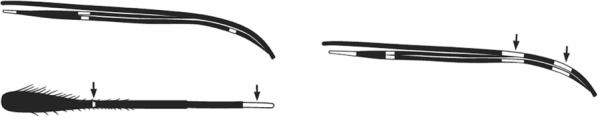
11. Maxillary palpus with 3 pale bands, subapical band broad and about equal in length to apical band ….7
12. Maxillary palpus either with 4 pale bands or if with 3 bands then subapical band much shorter than the apical band …9
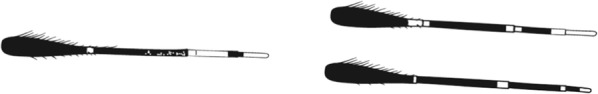
13. Wing, apart from costa, generally very pale, basal 0.5 of stems of veins 2 and 4 entirely pale ….*wellcomei*
  (in part)
14. Dark areas on wing greater than or about equal to pale areas, basal 0.5 of stems of veins 2 and 4 largely dark …8
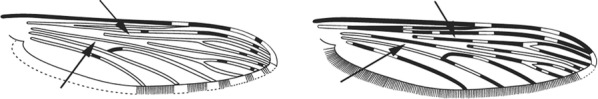
15. No pale fringe spots posterior to wing vein 3, stem of vein 5 pale except at fork and sometimes narrowly near base...*erepens*
16. Pale fringe spots present opposite all veins from wing apex to vein 5, stem of vein 5 broadly dark near base …*keniensis*
  (in part)
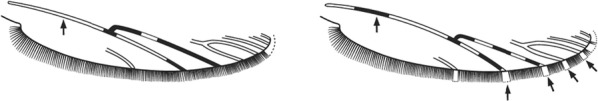
17. Wing vein 5 entirely dark except for a single pale spot on the upper branch …*fuscivenosus*
  (in part)
18. Wing vein 5 with extensive pale areas, upper branch of vein 5 with 2 pale spots ….10
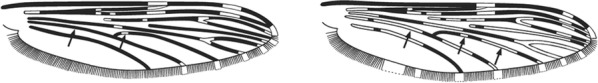
19. Hindtarsomeres 1 to 4 with distinct apical pale bands; scutum clothed with very narrow scales …*distinctus*
20. Hindtarsomeres 1 to 4 entirely dark or with a few pale scales at apices of 1 to 3; scutum scales broad …11
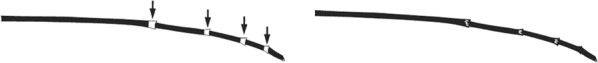
21. Median scutal scales yellowish or bronze, white elsewhere …*schwetzi*
  (in part)
22. Scutal scales white throughout ...*walravensi*
  (in part)
  *schwetzi*
  (in part)

### Section VII. Mosquitoes with maxillary palpus dark at apex or without distinct apical pale band; at least 1 pale spot on basal 0.5 of costa, pale scales not confined to costa and wing vein 1; legs not speckled, hindtarsomeres 4 and 5 not entirely pale; abdomen without laterally projecting tufts of scales

1. Maxillary palpus entirely dark or without distinct pale bands …2
2. Maxillary palpus with 3 pale bands ….5
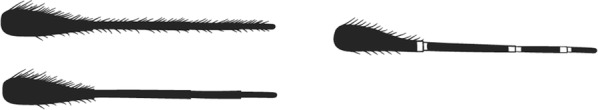
3. Small, pale brown species, pale patches on wing indistinct, basal 0.25–0.5 of costa entirely dark; head scales narrow and yellowish ….*azaniae*
  (in part)
4. Wing with well-contrasting pale and dark areas, basal 0.25 of costa with at least 1 pale area, even if narrow; head scales not so …3
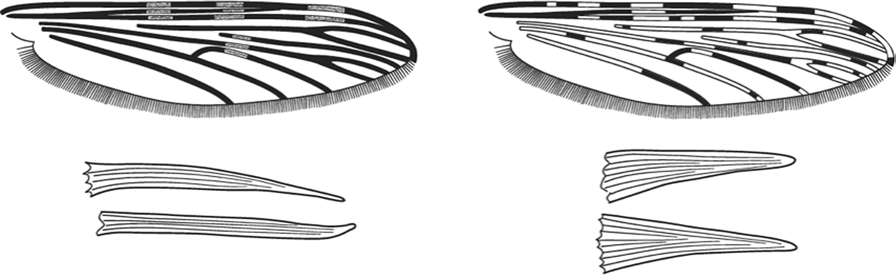
5. Costa with humeral pale spot, no subapical (preapical) pale spot on costa and vein 1 …*obscurus*
  (in part)
6. Costa without a humeral pale spot, subapical pale spot present on costa and vein 1 …4
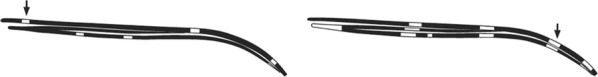
7. Wing with pale fringe spots opposite all veins except vein 6 …*jebudensis*
8. Wing with no pale fringe spots posterior to vein 3 ….*faini*
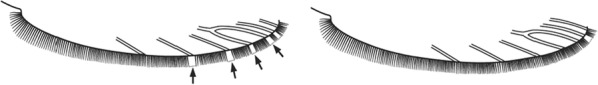
9. Wing generally pale, contrast between pale and dark areas, apart from costa and vein 1, poorly defined …*turkhudi*
10. Wing with well-contrasting pale and dark areas…6
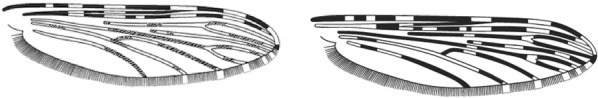
11. 2nd main dark area of wing vein 1 with 2 pale interruptions …7
12. 2nd main dark area of wing vein 1 with at most 1 pale interruption …8
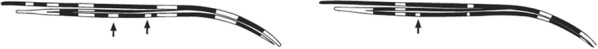
13. Pale bands on maxillary palpus very narrow, at apices of segments 2 to 4 and not overlapping the joints; upper branch of wing vein 5 with a single pale spot …*wilsoni*
  (in part)
14. Pale bands on maxillary palpus variable in width, distal 2 bands overlapping the joints; upper branch of wing vein 5 with 2 pale spots ...*rufipes*
  (in part)
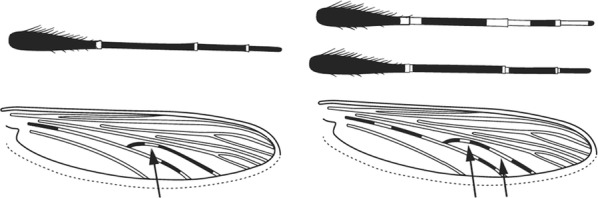
15. Wing, apart from costa and vein 1, predominantly dark, no pale spots on basal 0.25 of costa …9
16. Pale and dark areas on wing about equally distributed, humeral and presector pale spots present on costa …12
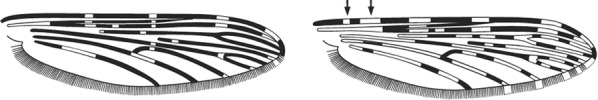
17. Wing vein 6 dark …*rageaui*
18. Wing vein 6 with proximal pale spot …10
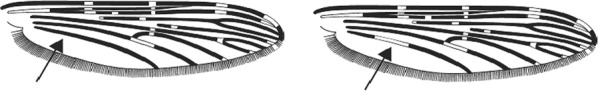
19. Basal 0.2 of wing vein 1 either dark or with a proximal pale patch not extending to base.…*smithii*
  (in part)
20. Basal 0.2 of wing vein 1 entirely pale ….11
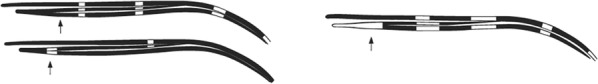
21. Wings scantily scaled, all wing scales very narrow.…*fontinalis*
22. Wings heavily scaled, upstanding scales moderately broad …*lovettae*
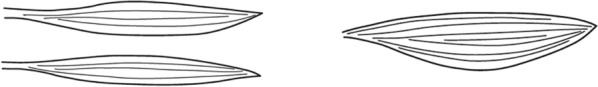
23. Basal pale band of maxillary palpus about equal to or slightly shorter than median band, broadly overlapping base of 3rd segment …*cinereus*
  (in part)
24. Basal pale band of maxillary palpus either much shorter than median band, scarcely overlapping base of 3rd segment, or both basal and median pale bands very narrow …13
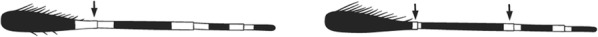
25. Base of costa pale …*multicolor*
  (north-east Africa only)
26. Base of costa dark ...*listeri*
  (southern Africa only)
  *azevedoi*
  (south-western Africa only)
  *seretsei*
  (Botswana only)
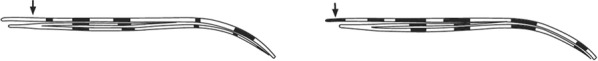

### Section VIII. Mosquitoes with smooth, 4-banded maxillary palpus, pale at apex; at least 1 pale spot on basal 0.5 of costa, pale scales not confined to costa and wing vein 1; legs not speckled, hindtarsomeres 4 and 5 not entirely pale; abdomen without laterally projecting tufts of scales

1. 3rd main dark area of wing vein 1 with a pale interruption.….2
2. 3rd main dark area without a pale interruption..3
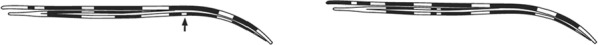
3. Abdominal terga clothed with yellowish scales; hindtarsomeres 1 to 4 with broad apical pale bands.…*christyi*
4. Abdominal terga without such scales; hindtarsomeres entirely dark or with a few pale scales at apices of hindtarsomeres 1 to 3 …*schwetzi*
  (in part)
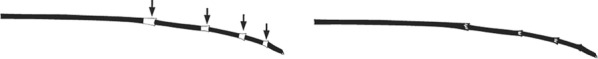
5. 2nd main dark area of wing vein 1 with 2 pale interruptions…*wilsoni*
  (in part)
6. 2nd main dark area of wing vein 1 with 1 pale interruption …4
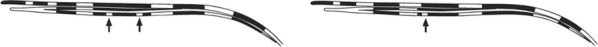
7. Pale bands on maxillary palpus broad, basal band overlapping base of 3rd segment …*cinereus*
  (in part)
8. Pale bands on maxillary palpus mostly narrow, basal band not overlapping base of 3rd segment ….5
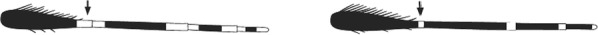
9. No pale fringe spots on wing posterior to vein 3; femora and tibiae inconspicuously speckled …..*vernus*
  (in part)
10. Pale fringe spots on wing present opposite veins posterior to vein 3, sometimes including vein 6; femora and tibiae not speckled.…6
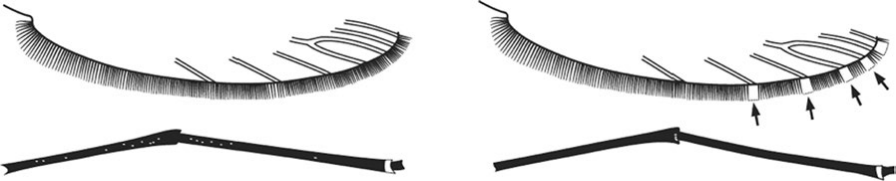
11. Stem of wing vein 5 pale, at and adjacent to the fork …..*garnhami*
  (in part)
12. Fork of wing vein 5 dark …7
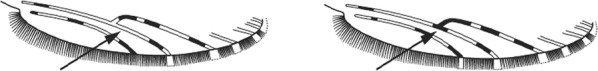
13. Wing length 4 mm or less; decumbent scutal scales not extending onto scutellum …*demeilloni*
  (Berg River form)
14. Wing length 4.4 mm or more; some decumbent scales present on scutellum as well as scutum …*carteri*
  (in part)

### Section IX. Mosquitoes with a pale interruption in 3rd main dark area (preapical dark spot) of wing vein 1 or this area entirely pale; at least 1 pale spot on basal 0.5 of costa, pale scales not confined to costa and vein 1; maxillary palpus with 3 pale bands, pale at apex; legs not speckled, hindtarsomeres 4 and 5 not entirely pale; abdomen without laterally projecting tufts of scales

1. 2nd and 3rd main dark areas of wing (median and preapical dark spots) absent from vein 1 …*wellcomei*
  (in part)
2. 2nd and 3rd main dark areas present on vein 1 …2
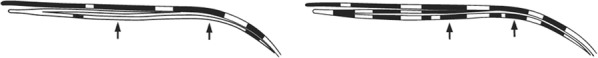
3. Hindtarsomere 5 entirely pale, hindtarsomere 4 with broad apical and basal pale bands ….*seydeli*
4. Hindtarsomere 5 entirely dark, hindtarsomere 4 with narrow apical and basal pale bands.…3
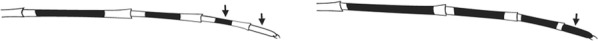
5. Upper branch of wing vein 5 with 1 pale spot, sometimes a vestigial 2nd pale spot …4
6. Upper branch of wing vein 5 with 2 well-developed pale spots.…5
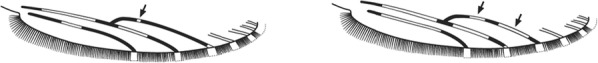
7. Pale fringe spot present opposite wing vein 6; foretarsomeres 1 to 4 with conspicuous basal and apical pale bands …*mortiauxi*
8. No pale fringe spot opposite wing vein 6; foretarsomeres 1 to 4 narrowly pale apically only …*berghei*
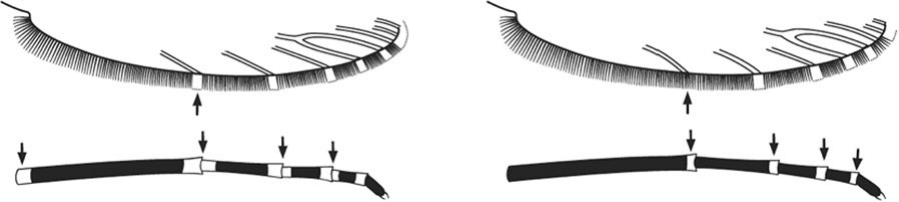
9. Subapical pale band on maxillary palpus very narrow, confined to apex of 3rd segment …6
10. Subapical pale band on maxillary palpus broad, overlapping apex of 3rd and base of 4th segment ….7
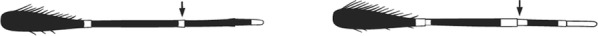
11. Wing with base of costa with 2 pale interruptions ….*brunnipes*
12. Basal 0.25 of costa entirely dark …*walravensi*
  (in part)
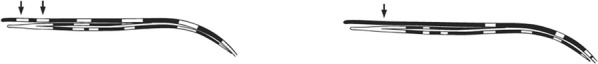
13. Hindtarsomeres either all dark or with pale bands on tarsomeres 1 and 2 only ….8
14. Hindtarsomeres 1 to 4 with well-marked apical pale bands …10
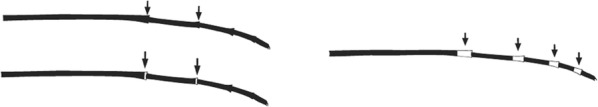
15. Scutal fossae and lateral areas of scutum above wing root (supraalar area) without scales …..*harperi*
16. Scutal fossae and lateral areas of scutum above wing root (supraalar area) with scattered or abundant broadish scales ….9
17. Subapical pale band on maxillary palpus about equal to or slightly shorter than apical band.…*njombiensis*
18. Subapical pale band on maxillary palpus much narrower than apical band …*walravensi*
  (in part)
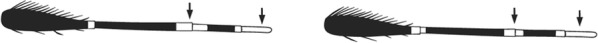
19. Apical pale bands on hindtarsomeres 1 to 4 very broad, at least twice the apical width of the tarsomeres.…*austenii*
20. Hindtarsomeres 1 to 4 with narrow pale bands, as long or shorter than the width of the tarsomeres …11
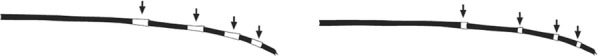
21. Wing vein 3 largely dark or broadly dark at either end; scutal scales very narrow and golden …*gibbinsi*
  (in part)
22. Wing vein 3 narrowly dark at ends; scutal scales various …12
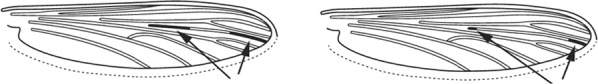
23. Scutal scales as in A ……*hargreavesi*
  Scutal scales as in B …*mousinhoi*
  Scutal scales as in C …*marshallii*
  *letabensis*
  *kosiensis*
  *hughi*
24. Scutal scales as in D …*gibbinsi*
  (in part)
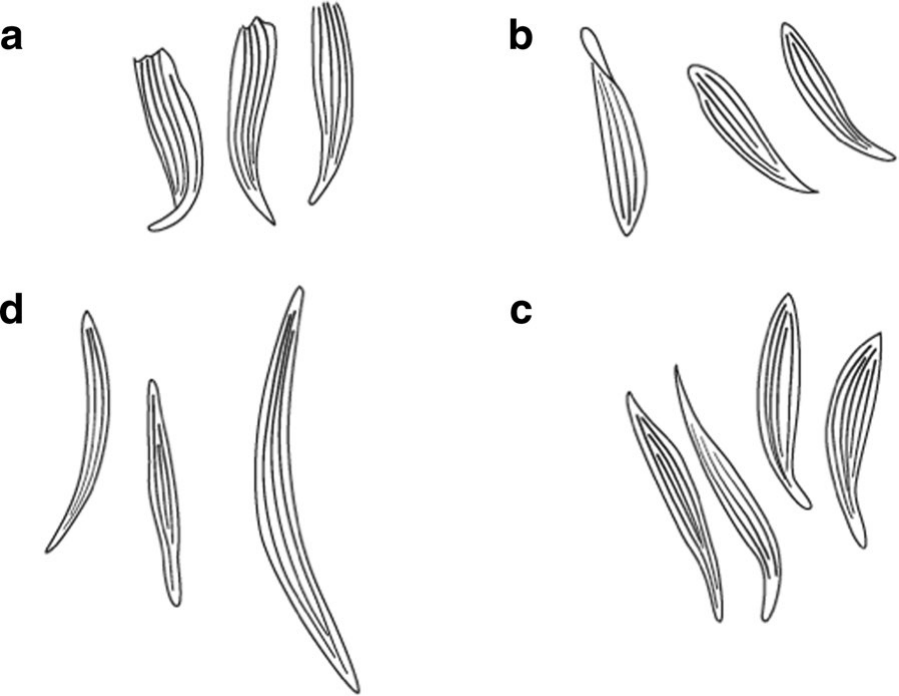

### Section X. Mosquitoes with upper branch of wing vein 5 with 2 pale spots, no pale interruptions in 3rd main dark area of vein 1, at least 1 pale spot on basal half of costa, pale scales not confined to costa and vein 1; maxillary palpus with 3 pale bands or less, pale at apex; legs not speckled, hindtarsomeres 4 and 5 not entirely pale; abdomen without laterally projecting tufts of scales

1. Maxillary palpus with only apical pale band …*gabonensis*
  Maxillary palpus with 3 pale bands.….2
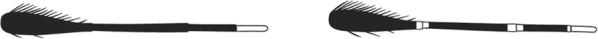
2. Subapical pale band on maxillary palpus broad, about equal to or longer than apical dark band …3
3. Apical dark band much longer than subapical pale band ...17
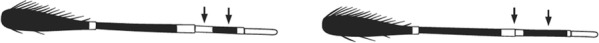
4. Apical pale band on hindtarsomere 4, and sometimes on hindtarsomeres 2 and 3, extending onto bases of succeeding tarsomeres ….4
5. Bases of hindtarsomeres dark …7
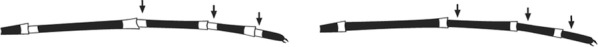
6. 2nd main dark area on wing vein 1 with 2 pale interruptions; bases of hindtarsomeres 4 and 5 broadly or narrowly pale …*rufipes*
  (in part)
7. 2nd main dark area on wing vein 1 with 1 pale interruption; bases of hindtarsomeres 4 and 5 at most narrowly pale …5
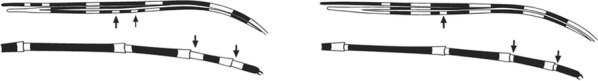
8. Base of costa with 1 pale interruption, 3rd main dark area on costa and vein 1 much broader than subcostal pale spot …*domicolus*
9. Base of costa with 2 pale interruptions, 3rd main dark area equal to or narrower than subcostal pale spot …6
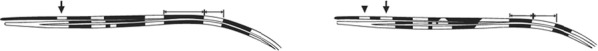
10. Pale fringe spot present opposite wing vein 6 …*lloreti*
11. No pale fringe spot opposite vein 6 …*barberellus*
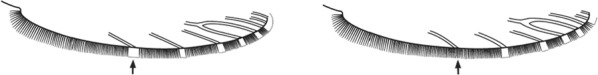
12. Apices of hindtarsomeres 3 and 4 dark or at most with a few pale scales …8
13. Apices of hindtarsomeres 1 to 3 and sometimes 4, distinctly pale banded …13
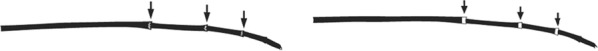
14. Base of costa with 1 or no pale interruption …..9
15. Base of costa with 2 pale interruptions…10
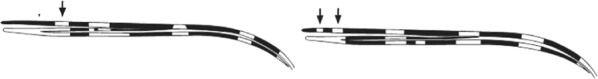
16. Wing vein 6 either with pale fringe spot or with pale scales at apex of vein …*brucei*
  (in part)
17. Wing vein 6 without pale fringe spot and no pale scales at apex …*rivulorum*
  (in part)
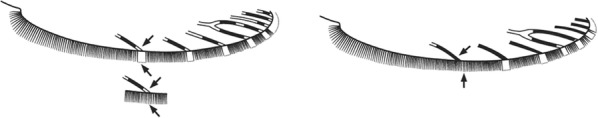
18. Scutal scales fairly broad, extending over whole scutum and onto scutellum …*carteri*
  (in part)
19. Scutal scales variable, but decumbent scales confined to at most anterior 0.66 of scutum …11
20. Very small species, wing length 2.8 mm or less …*brucei*
  (in part)
21. Small or moderate species, wing length 2.9 mm or more …12
22. Hindtarsomeres entirely dark; preaccessory dark spot on wing vein 1 usually absent …*freetownensis*
23. Hindtarsomeres 1 and 2 narrowly but distinctly pale apically; preaccessory dark spot present on wing vein 1 …*demeilloni*
  (in part)
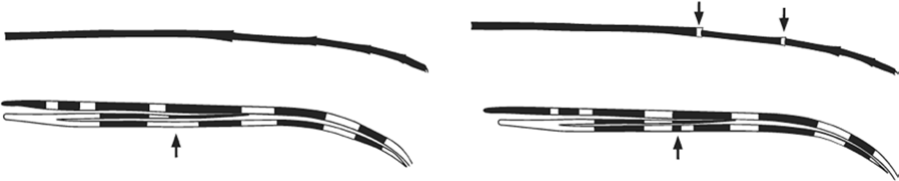
24. 3rd main dark area of costa equal to or shorter than subapical pale spot …*flavicosta*
  (in part)
25. 3rd main dark area much longer than subapical pale spot …14
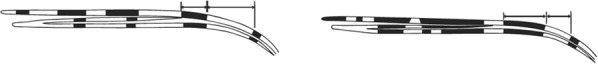
26. Scutal scales broadish and white, only slightly less dense on posterior 0.33 of scutum than anteriorly, and extending onto scutellum …*flavicosta*
  (in part)
27. Scutal scales on posterior 0.33 of scutum scanty, narrow and yellowish-brown …15
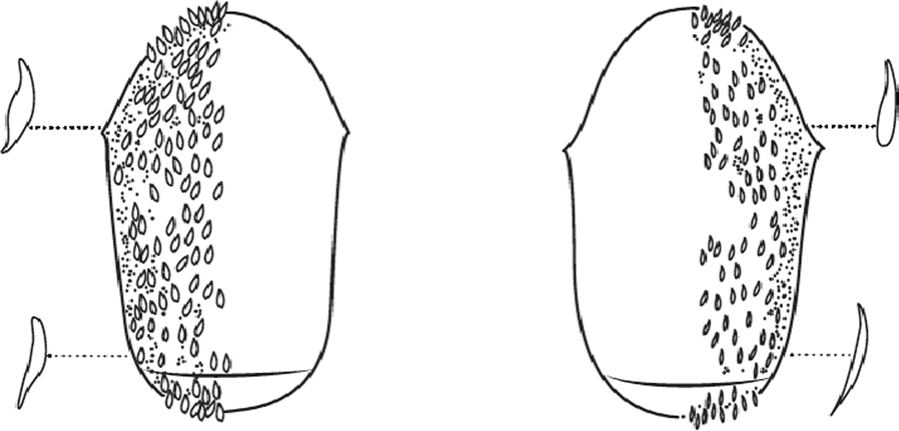
28. Moderate-sized species, wing length more than 3.2 mm …*keniensis*
  (in part)
29. Small species, wing length 3.0 mm or less …16
30. Foretarsomere 4 dark or indistinctly pale at apex; wing usually without pale fringe spot opposite vein 6 …..*moucheti*
31. Foretarsomere 4 with well-marked apical pale band; wing with fringe spot opposite vein 6 …*bervoetsi*
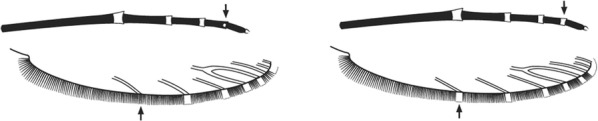
32. 3rd main dark area of costa equal to or shorter than subapical pale spot …*flavicosta*
  (in part)
33. 3rd main dark area of costa much longer than subapical pale spot ….18
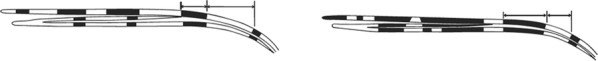
34. Wing with fork of vein 5 pale..….*garnhami*
  (in part)
35. Wing with fork of vein 5 dark …19
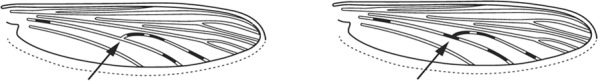
36. Base of costa with 2 pale interruptions …*demeilloni*
  (in part)
37. Base of costa with 1 or no pale interruption …20
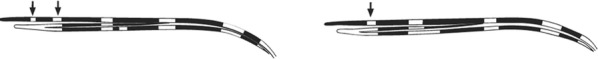
38. Small species, wing length about 2.4–3.3 mm …*rivulorum*
  (in part)
39. Small or moderate-sized species, wing length 2.9–4.2 mm …*demeilloni*
  (in part)

### Section XI. Mosquitoes with upper branch of wing vein 5 with 1 pale spot, no pale interruptions on 3rd main dark area of vein 1, at least 1 pale spot on basal 0.5 of costa, pale scales not confined to costa and vein 1; maxillary palpus with 3 pale bands or less, pale at apex; legs not speckled, hindtarsomeres 4 and 5 not entirely pale; abdomen without laterally projecting tufts of scales

1. Maxillary palpus with only apex pale…2
  Maxillary palpus with 3 pale bands.….5
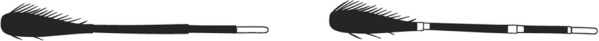
2. Base of costa with large (presector) pale spot, base of vein 1 pale …3
  Base of costa dark or with small pale spot, base of vein 1 dark …4
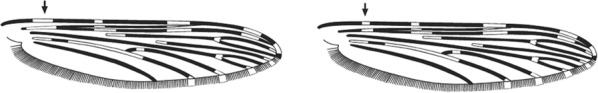
3. Lower branch of wing vein 2 and upper branch of vein 4 with distinct pale spots …*carnevalei*
4. These veins dark ….*ovengensis*
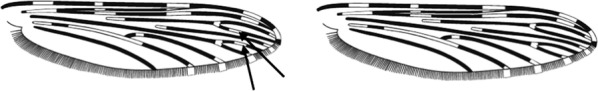
5. Subapical pale spot on costa and wing vein 1 about as long as apical dark spot, fringe spots present opposite veins 3, lower branch of 4 and both branches of 5 …*nili*
  Congo form
6. Subapical pale spot shorter, usually much shorter, than apical dark spot, no pale fringe spot opposite upper branch of vein 5 …*nili*
  *somalicus* 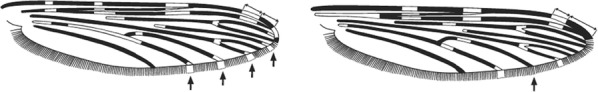

7. Hindtarsomeres 1 to 4 with pale bands overlapping the joints, at least hindtarsomere 5 pale basally …*longipalpis*
8. Pale banding on hindtarsomeres narrow and apical only …6
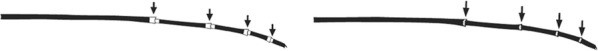
9. Preaccessory dark spot on wing vein 1 about twice as long as pale spot on either side of it …*fuscivenosus*
  (in part)
10. Preaccessory dark spot absent or, if present, shorter or only slightly longer than adjoining pale spots …7
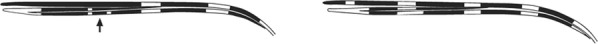
11. Basal area of wing vein 1 proximal to 1st main dark area, pale with a broad dark spot …*culicifacies*
12. Basal area of wing vein 1 entirely pale…8
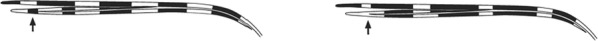
13. Subapical pale band on maxillary palpus longer than or equal to apical dark band **AND** 3rd main dark area of costa and vein 1 equal to or shorter than subapical pale spot ….*aruni*
14. Subapical pale band on maxillary palpus much shorter than apical dark band, **OR** 3rd main dark area longer than subapical pale spot…9
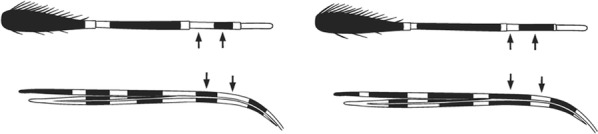
15. Moderate-sized species, wing length more than 3.3 mm …*demeilloni*
  (in part)
16. Small species, wing length 3.2 mm or less …10
17. Tip of wing vein 6 with a few pale scales, sometimes with fringe spot present …*parensis*
18. Tip of wing vein 6 dark with no fringe spot…*funestus* group
  *sergentii*
  *demeilloni*
  (in part; mainly highlands)
  *cameroni*
  (extreme S. Africa only)
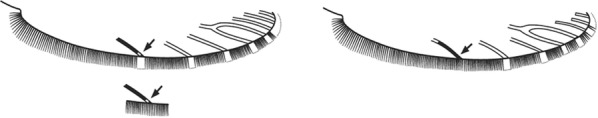

## Data Availability

Not applicable.
